# Alarm fatigue in healthcare: a scoping review of definitions, influencing factors, and mitigation strategies

**DOI:** 10.1186/s12912-025-03369-2

**Published:** 2025-06-20

**Authors:** Elizabeth Anna Mathilde Michels, Stephen Gilbert, Iulia Koval, Magdalena Katharina Wekenborg

**Affiliations:** 1 Else Kröner Fresenius Center for Digital Health, Faculty of Medicine, Technical University Dresden, Fetscherstraße 74, 01307 Dresden, Germany; 2 Faculty of Psychology, Technical University Dresden, Zellescher Weg 17, 01069 Dresden, Germany

**Keywords:** Alarm fatigue, Patient safety, Nurses, Healthcare quality improvement, Well-being at work

## Abstract

**Background:**

Alarm Fatigue is recognized as a significant risk to both patient safety and the well-being of healthcare professionals (HCPs). However, it remains an underexplored phenomenon, further complicated by the lack of a harmonized definition. This review aims to (1) propose a harmonized definition; (2) identify operationalization methods; (3) summarize influencing factors; (4) examine consequences; and (5) outline potential strategies for reducing Alarm Fatigue.

**Methods:**

This scoping review was conducted following the *Preferred Reporting Items for Systematic Reviews and Meta-Analyses extension for Scoping Reviews* (PRISMA-ScR). A systematic search was conducted in PubMed, CINAHL, MEDLINE, and Google Scholar using keywords related to Alarm Fatigue, covering the literature published up to April 2024. Publications were included if they addressed at least one of the five review objectives. Extracted data covered definitions, operationalization methods, influencing factors, consequences, and mitigation strategies.

**Results:**

A total of 32 publications were included. Definitions varied, but most described Alarm Fatigue as a phenomenon in which repeated exposure to frequent or non-actionable alarms leads to sensory overload, emotional strain, and a gradual desensitization or reduced responsiveness among HCPs, increasing the risk of delayed or inadequate alarm responses and compromising patient safety. Self-report questionnaires and observational methods were most frequently used tools for operationalization, while physiological and lab-based approaches were rare. Alarm overload, psychosocial work conditions, and individual traits have been identified as factors that may increase the likelihood of Alarm Fatigue. In turn, Alarm Fatigue was linked to delayed alarm responses, communication breakdowns, and increased stress and burnout among HCPs. Identified strategies for reducing Alarm Fatigue included training programs, technical improvements (e.g., alarm customization), protocol adjustments, and broader organizational interventions.

**Conclusions:**

The review highlights Alarm Fatigue as a complex and clinically relevant issue. By proposing a harmonized definition and mapping key findings across five domains, it offers a structured foundation for future research. Standardized definitions and measurement tools, along with targeted multi-level interventions, are essential for addressing Alarm Fatigue and improving both patient safety and working conditions in healthcare.

**Trial registration:**

The review was preregistered on the website https://aspredicted.org with the preregistration number #169,578.

**Clinical trial number:**

Not applicable.

**Supplementary Information:**

The online version contains supplementary material available at 10.1186/s12912-025-03369-2.

## Rationale

Alarm Fatigue is one of the most significant technological hazards, as it negatively affects patient safety [1], healthcare professionals (HCPs), as well as hospitals and society [2, 3]. The absence of a consensual definition of Alarm Fatigue remains a challenge. Nevertheless, there is general consensus that Alarm Fatigue represents a form of alarm desensitization, caused by factors such as high number of false alarms, the poor design and inadequacy of systems and the complexity of monitoring systems including the confusion in identifying what has triggered the alarm [4]. The trend of increased use of alarms in hospital medical devices could further exacerbate this problem. At the same time, if adequately addressed in the digital transformation of hospitals, there is a potential to reduce Alarm Fatigue through better design of devices and their interoperability, thereby enhancing patient safety and improving the working conditions of HCPs.

To understand and address Alarm Fatigue effectively, a clear conceptual and empirical foundation is needed. However, the current literature remains scattered and lacks a universally accepted definition, which presents a significant challenge for both research and clinical practice. Although previous reviews have attempted to summarize the literature, they mostly focus on specific aspects such as reduction strategies [5–7], nurses’ work [8], or isolated causes and consequences [3,9]. We aim to address this research gap with the present scoping review by conducting a comprehensive analysis of the overall phenomenon of Alarm Fatigue. A scoping review was considered particularly suitable for this work, as it allows for systematically mapping the breadth and nature of the existing literature on Alarm Fatigue, an emerging and conceptually diffuse field. Following the updated guidance of the Joanna Briggs Institute, this method enabled the identification of key concepts, research gaps, and the range of available evidence, without restricting inclusion to specific study types or requiring formal quality appraisal [10].

**Fig. 1:**
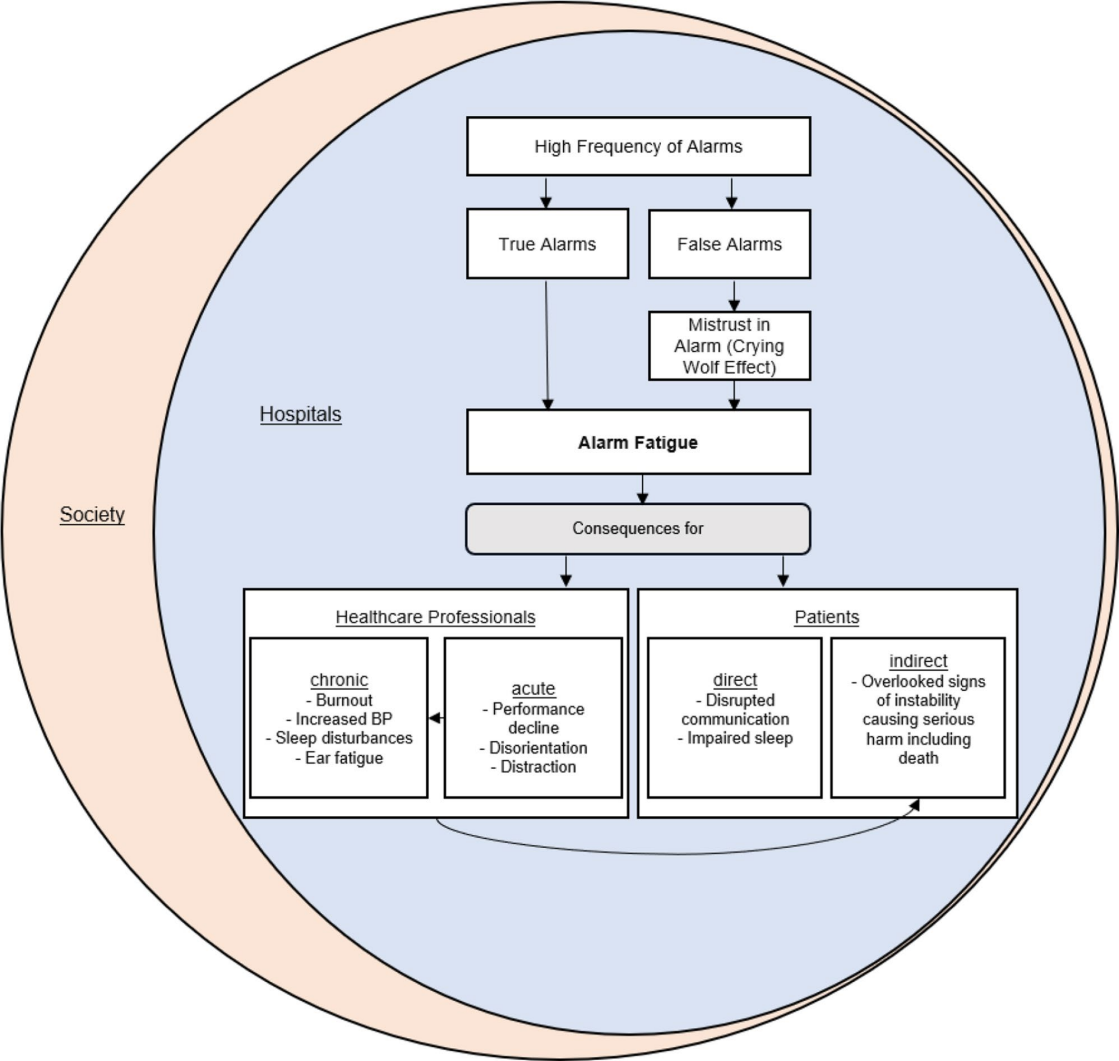
Alarm fatigue represented through a chain figure

This review focuses on the center of Fig. 1, which illustrates a systematic approach.

Previous research shows that an excessive number of true alarms, as well as a high number of false or non-actionable alarms, can trigger Alarm Fatigue (as shown in the upper part of Fig. 1). A high frequency of false alarms creates distrust in the alarm system, leading to a gradual wearing down of HCPs confidence in the utility of the alarm, and eventually leading to disregard of the alarm, even when it later may be signaling a genuine medical problem – a phenomenon known as the *‘Crying Wolf’* effect [11]. Alarm Fatigue has both direct and indirect consequences for patient safety (as shown in the lower part of Fig. 1). Direct consequences for patient safety are, for example, impaired patient sleep resulting from an excessive number of alarms. Indirect consequences for patient safety arise from the effects of Alarm Fatigue on HCPs. These indirect consequences may be due to consequences of Alarm Fatigue in HCPs like performance deterioration [7]. Additionally, chronic effects from prolonged Alarm Fatigue can cause a sustained burden on HCPs, which itself poses a significant risk to both HCPs health [12] and patient safety [13, 14].

At the same time, Alarm Fatigue generates high costs for the healthcare system [2, 3]. These include direct costs, such as higher healthcare expenses from longer hospital stays [15], and indirect costs, such as increased sick leave and a resulting lack of specialists due to chronic work-related stress and burnout among HCPs [16–18]. Both the increased healthcare costs and high rates of sick leave among HCPs have a significant impact on society.

### Objectives

Background research shows that Alarm Fatigue poses a serious threat to patient safety, HCPs health, hospitals, and society as a whole. To address this issue, a comprehensive understanding is required, covering the conceptualization and measurement of Alarm Fatigue, its direct causes, and additional factors that may influence or moderate its emerge, such as individual characteristics or organizational context. This review aims to make a significant contribution by: (1) proposing a harmonized definition of Alarm Fatigue, integrating the descriptions from the publications included in this review; (2) identifying the methods used to operationalize Alarm Fatigue in these studies; (3) summarizing empirical findings on the influencing factors of Alarm Fatigue, including psychosocial working conditions and individual factors; (4) examining the consequences of Alarm Fatigue for patient safety and HCPs; and (5) briefly discussing potential strategies for reducing Alarm Fatigue.

## Methods

### Protocol and registration

This scoping review was conducted in accordance with the *Preferred Reporting Items for Systematic Reviews and Meta-Analyses extension for Scoping Reviews* (PRISMA-ScR) guidelines [19] to ensure methodological rigor, transparency, and reproducibility. The review protocol was preregistered on https://aspredicted.org (registration number #169578).

### Eligibility criteria

For the systematic search strategy, inclusion and exclusion criteria were defined to include a representative number of publications. The criteria were defined using the PCC acronym, as briefly summarized in Table 1.

**Table 1:** Inclusion criteria based on the PCC acronym for literature search and selection

PCC acronym	Inclusion criteria
Population	▪ Nurses, physicians, or other healthcare providers (> 18 years) regularly exposed to monitoring alarms ▪ Other participants (> 18 years; for laboratory studies)
Concept	Individual studies that include at least one of the following aspects: ▪ Definition of the term “Alarm Fatigue” ▪ Operationalization of Alarm Fatigue ▪ Effects of repeated alarm exposure on patient safety ▪ Psychological and physical effects of repeated alarm exposure on healthcare staff ▪ Proposed solutions for reducing Alarm Fatigue Systematic Reviews: ▪ Include studies that meet the above criteria
Context	▪ The context is international ▪ Language: English ▪ Hospitals, intensive care units, healthcare centers, laboratories ▪ Influence of workload and shift work on alarm responses ▪ Influence of individual factors (e.g., age, gender, work experience, personality traits, etc.) on Alarm Fatigue

### Inclusion criteria

Publications were eligible for inclusion if they addressed at least one of the five objectives of this review: defining the concept of Alarm Fatigue, its operationalization, examining potential influencing factors, investigating consequences for patient safety and HCPs, or proposing strategies for its reduction. To be included, publications had to investigate healthcare professionals aged 18 years or older who are regularly exposed to monitoring alarms in clinical settings including hospitals, intensive care units, health centers, or laboratories. Experimental studies conducted under controlled laboratory conditions were also included if they involved participants from other professional backgrounds, as they allow for high experimental control and standardized assessment of Alarm Fatigue. However, we acknowledge that the use of non-HCP samples in these studies limits the generalizability of findings to clinical practice.

Furthermore, only publications published in English were considered, with no restrictions on study design or country of origin.

No exclusion criteria were applied regarding the time of publication or study execution, allowing the inclusion of research on both early digitization processes and the most recent developments in digital transformation. Moreover, there were no restrictions regarding the geographical setting to ensure that publications from regions with varying levels of digital development could be considered.

### Information sources

The literature was identified through several information sources, selected to ensure comprehensive coverage of the topic. These included PubMed, CINAHL, MEDLINE, and Google Scholar. Additionally, reference lists of included publications were manually screened, and additional records were identified via Google Scholar.

### Search

The literature search followed a three steps strategy based on the Population, Concept, Context (PCC) framework [20]. First, a preliminary search was conducted in PubMed and Google Scholar to identify relevant keywords and indexing terms. These insights were used to refine the search strategy (see Supplementary Table 1 in supplementary materials). Second, a systematic search was performed in PubMed, CINAHL, MEDLINE, and Google Scholar using keywords related to Alarm Fatigue with Boolean operators. Third, a manual search of reference lists and additional records via Google Scholar was conducted to identify further eligible publications. The search covered literature published up to April 2024.

All search results were imported into a reference management software [21], and duplicates were removed. Titles and abstracts of the remaining records were screened for eligibility based on the predefined criteria. Full texts of potentially relevant publications were assessed independently by the review team, with disagreements resolved through discussion.

### Data extraction

Data extraction was conducted in accordance with the PRISMA-ScR guidelines [19]. The following data were extracted: authors, year of publication, title, journal, study design, country, definition of alarm fatigue, sample, operationalization of alarm fatigue, proposed solutions for reducing alarm fatigue, individual influencing factors of alarm fatigue, and key study findings. The collected data were summarized in Supplementary Table 2 (see supplementary materials).

### Critical appraisal of individual sources of evidence

No critical appraisal of the included publications was conducted, in line with guidance, which states that scoping reviews aim to map the breadth of available evidence rather than assess methodological quality [10].

### Data synthesis

The findings from the included studies were synthesized using a narrative approach, structured around the five aims of this scoping review. Specifically, we analyzed the literature to (1) propose a harmonized definition of Alarm Fatigue, (2) identify operationalization methods, (3) summarize influencing factors, (4) examine consequences, and (5) discuss potential strategies for reducing Alarm Fatigue. This framework guided the organization and interpretation of the data.

## Results

### Selection of sources of evidence

**Fig. 2:**
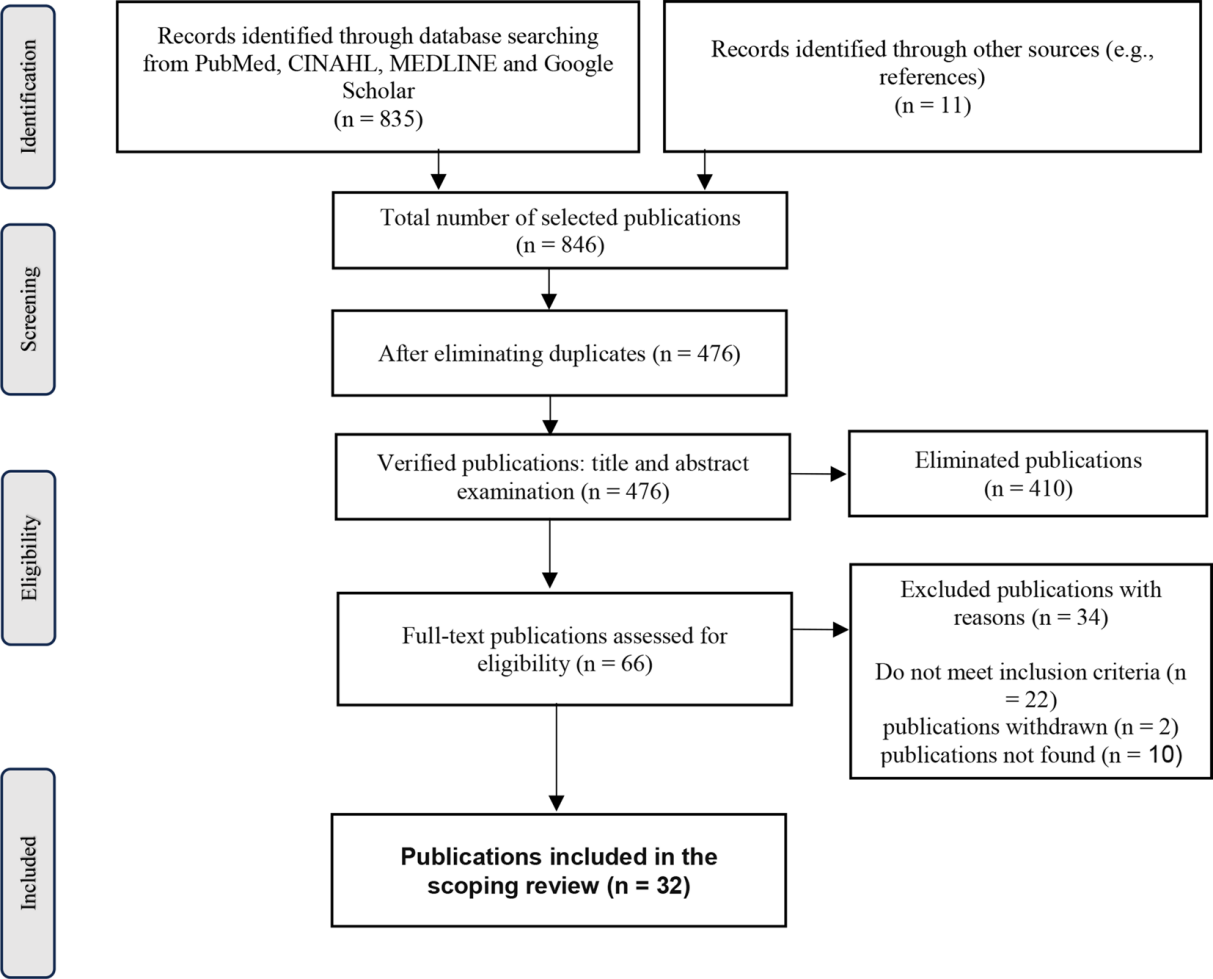
PRISMA Flow diagram of the study selection process (Preferred reporting Items for systematic reviews and meta-analyses)

As shown in the PRISMA flow diagram (Fig. 2), the database search yielded 846 records. After removing 370 duplicates, 476 records remained for screening. Based on title and abstract screening, 410 records were excluded. Of the 66 full-text articles assessed for eligibility, 34 were excluded: 22 did not meet the inclusion criteria, 2 had been retracted, and 10 could not be retrieved. A total of 32 publications were included in the final review.

### Characteristics of studies

The 32 included publications were published between 2012 and 2024, with 72% published in the last six years [4–6, 8, 9, 16–18, 22–36]. Regarding geographic distribution, 15 publications (46.88%) originated from Asia [6, 9, 16, 17, 22–24, 26–28, 30, 32, 33, 36, 37], 8 (25%) from North America [1, 4, 7, 25, 31, 35, 38, 39], 6 (18.75%) from Europe [3, 5, 8, 29, 40, 41], 2 (6.25%) from South America [34, 42], and 1 study (3.13%) from Africa [18].

Following the classification proposed by Lapeña and Peh [43], 23 publications were categorized as *Primary or Original Research Articles* (13 cross-sectional publications [8, 16, 17, 22–24, 26–28, 32–34, 40], 2 observational studies [38, 42], 2 descriptive researches [25, 37], and 6 with other research designs [30, 31, 35, 36, 39, 41] and 9 as *Secondary or Review Articles* (4 integrative reviews [1, 4, 6, 32], 3 systematic literature reviews [5, 9, 29], 1 narrative review [7], and 1 scoping review [3].

### Study participants and settings

The included publications were conducted in a variety of healthcare contexts, including hospitals, intensive care units (ICUs), and critical care areas. With regard to professional background, the majority of study samples consisted nurses (*N* = 11; [8, 9, 16, 17, 22, 23, 26–28, 30, 32, 33, 35–37, 40], followed by mixed-profession samples involving nurse, physician, technician, and physiotherapist participants (*N* = 5; [5, 24, 25, 34, 38]. In addition, several experimental studies were conducted under controlled laboratory conditions, using non-clinical participants such as university students [31, 41]. Sample sizes varied widely, with the largest being *N* = 400 participants [8] and the smallest being *N* = 10 participants [41], with *M* = 163,45 and *SD* = 136,08.

### Synthesis of results

All included publications addressed one or more of the five predefined review objectives, namely: (1) proposing a harmonized definition; (2) identifying operationalization methods; (3) summarizing influencing factors; (4) examining consequences; and (5) discussing potential strategies for reducing Alarm Fatigue. The following sections present results organized by these objectives.

#### Definition of alarm fatigue

The first goal of this review was to provide a harmonized definition of Alarm Fatigue by transforming the various descriptions found in the included publications into a more formalized and interrelated structure. However, after evaluating the publications, this appears difficult, as a significant portion of the publications either do not provide a comprehensive definition or completely omit formal definitions, instead offering loose descriptions. Despite the differences across publications, the majority of the included publications provide descriptions of Alarm Fatigue that highlight several common themes, summarized as follows: Alarm Fatigue primarily affects individuals working in clinical and safety-critical environments like ICUs and emergency areas [26, 41, 44] or hospitals [45], where patients rely on various monitoring devices [32]. It occurs when HCPs are exposed to an excessive number of alarms [1, 23, 40], with some publications noting that frequent false alarms contribute significantly to its development [24, 37]. Alarm fatigue is associated with sensory overload [23], diminished responsiveness to alarms [15, 23, 24, 40], and desensitization to alarm signals [27], often accompanied by emotional strain [26]. As a result, HCPs may delay or inadequately respond to alarms [7, 29, 38, 41], develop skepticism toward the alarm system [45], or deactivate alarms [1, 7, 15], and reduce their volume [33]. Based on the recurring themes identified across the included publications, we propose the following working definition of Alarm Fatigue: Alarm Fatigue is a phenomenon in healthcare settings in which repeated exposure to frequent or non-actionable alarms leads to sensory overload, emotional strain, and a gradual desensitization or reduced responsiveness to alarms among healthcare professionals. This in turn increases the risk of delayed or inadequate alarm responses, with potentially serious consequences for patient safety.

#### Operationalization of alarm fatigue

In line with the second aim of this review, we identified methods used in the included publications to measure Alarm Fatigue. A total of 27 distinct methods were identified, as detailed in Table 2.

**Table 2:** Operationalizing alarm fatigue

Main categories	Operationalizing alarm fatigue	Sources	Results
1. Questionnaires and scales to measure Alarm Fatigue and related constructs (*n* = 16)	The Nurses’ Alarm Fatigue Questionnaire (Torabizadeh et al., 2017)	Ali Al-Quraan et al., 2023; Alkubati et al., 2024; Bourji et al., 2020; Ding et al., 2023; Ergin et al., 2023; Ilter & Ovayolu, 2023; Lewandowska et al., 2023; Regmi et al., 2023; Yahyaei et al., 2023	9
	Questions about monitoring devices and alarm management	Bourji et al., 2020	1
	Clinical Alarm Survey (Clinical Alarms Task Force, 2007)	Claudio et al., 2021; Deb & Claudio, 2015	2
	Self-developed questionnaire for assessing Alarm Fatigue	Cho et al., 2016; Dehghan et al., 2023	2
	Alarm Fatigue Scale (Kahraman et al., 2020)	Gündoğan & Erdağı Oral, 2023	1
	A Validated Full-Structured Nurse Alarm Fatigue Questionnaire (Yin, Z., 2021)	Nyarko et al., 2023	1
2. Observations and direct measurements (*n* = 6)	Direct measurements	Carcereri de Oliveira et al., 2018; Cho et al., 2016; Claudio et al., 2021; Deb & Claudio, 2015; Stiglich et al., 2023; Storm & Chen, 2021	6
3. Measurements in laboratory experiments (*n* = 4)	Tasks in the laboratory	Cobus et al., 2018; Nagrecha & Baldwin, 2022	2
	n-back-task	Cobus et al., 2018; Nagrecha & Baldwin, 2022	2
4. Other instruments and methods (*n* = 1)	Semi-structured interviews	Movahedi et al., 2023	1

The most common approach to assessing Alarm Fatigue involved questionnaires (*N* = 16), with “The Nurses’ Alarm Fatigue Questionnaire” [46] being most frequently used, appearing in nine studies. Six studies employed observational methods, such as monitoring behavior during alarms [37], measuring alarm frequency [14, 30, 38], response time [15, 25, 35, 38], and the ratio of true to false alarms ratio [44]. Additionally, one study measured sound levels in decibels [15].

Laboratory designs were used in only two studies, which applied four distinct methods. Cobus et al. developed a simulated nursing environment to replicate the cognitive, physical, and precision demands of nursing shifts [41]. Participants completed tasks such as removing plastic items from cavities using tweezers without touching the edges, guiding a wand along a wire without contact, and refilling syringes with precise amounts of water - all requiring precision and providing feedback for errors. This setup validated a novel alarm system using light patterns displayed on head-mounted devices. Participants performed these tasks while responding to alarms and subsequently rated the urgency of each alarm. Similarly, Nagrecha et al. used a dual-task laboratory method to mimic critical care demands. Participants responded to real alarms and ignored false ones while performing a working memory task (a 2-back task) on separate monitors [31]. In both studies, participants had to manage a primary task while responding to alarms, simulating the multi-tasking challenges inherent in healthcare settings. Other identified instruments included semi-structured interviews.

#### Influencing factors

In line with our third aim, which was to identify factors influencing the occurrence of Alarm Fatigue, 20 of the 32 selected publications explored these factors. These factors were grouped into two main categories: environmental factors and individual factors.

### Individual factors

Several studies examined **demographic factors** such as gender, age, and marital status, though findings remain inconclusive, particularly with respect to gender [23, 28, 35]. One study suggests that men, individuals aged 21–40, and married people may experience higher levels of Alarm Fatigue [23], while others found higher Alarm Fatigue levels among female nurses compared to their male colleagues [9, 35, 36].

With respect to **professional factors**, findings regarding the influence of years of experience and education level on Alarm Fatigue were also mixed. One study reported higher Alarm Fatigue among HCPs with over ten years of experience [23], while others found that more experience was associated with lower levels of Alarm Fatigue [27, 32]. Similarly, while some studies found that nurses with higher educational levels reported lower levels of Alarm Fatigue [9, 26], others found no significant correlation between Alarm Fatigue and education [22, 24, 40]. Moreover, Alarm Fatigue was observed across various healthcare professions including both, nurses and physicians [28].

The findings regarding **personality traits** are coherent. However, it is important to note that these traits were examined in only two studies [25, 47]. Extraverted individuals, who are highly responsive to external stimuli, may be more prone to Alarm Fatigue, as they tend to react to every alarm, including irrelevant ones [38, 45]. Nonetheless, their assertiveness and high activity levels can help them to manage alarms more effectively [25, 47]. Neuroticism, characterized by emotional instability, is linked to greater anxiety and irritability, increasing susceptibility to Alarm Fatigue [25, 47]. In contrast, conscientiousness, associated with organization and responsibility, appears to reduce Alarm Fatigue, as conscientious individuals tend to plan and create routines that minimize desensitization [25, 47]. Agreeable individuals, motivated by empathy, may respond more frequently to alarms, potentially heightening their stress [38]. Lastly, individuals high in openness, due to their curiosity and adaptability, may handle repeated alarms more effectively, according to the authors of the papers [25, 47].

The results regarding psychological and health-related factors, although based on only three studies [17, 32, 39], indicate bidirectional links between Alarm Fatigue and stress, exhaustion, depersonalization, anxiety, reduced personal fulfillment, sleep deprivation, substance use, and personal health [17, 32, 39].

### Environmental factors

The results regarding **work environment and workload**, while more extensively studied, remain inconclusive [1, 8, 9, 23–26, 28, 35, 40]. High cognitive load, long hours, and noisy environments have been linked to Alarm Fatigue in some studies [1, 8, 25, 40], while others found no significant links between average weekly workload and Alarm Fatigue [35]. Similarly, the impact of shift work on Alarm Fatigue is inconsistent, with some studies reporting higher Alarm Fatigue during night shifts [9, 24, 25] and others during day shifts [23, 28].

The results regarding **training**,** protocols and team dynamics**, though based on only two studies, provide more conclusive insights [3, 9]. Inadequate training, unclear protocols, and poor team communication were identified as factors that increase Alarm Fatigue [3]. Insufficient training and unclear communication structures were reported in the included studies in connection with delayed alarm response or decision-making challenges [9].

#### Consequences for patient safety and HCPs

The publications included in this review highlight the significant impact of Alarm Fatigue on patient safety and HCPs. With respect to HCPs, publications show that Alarm Fatigue deteriorates performance [38, 40, 48], and manifests in various acute symptoms, including disorientation, distraction [39, 48], impaired communication, reduced concentration [40], and compromised decision-making [40], leading to unnecessary work [3, 40] and frequent interruptions [3, 40]. Chronic symptoms reported included frustration, burnout [40], elevated blood pressure [40], chronic stress [40], sleep disturbances [40], and auditory fatigue [40]. However, one study did not find significant associations with burnout [35]. Psychological consequences such as self-blame and post-traumatic stress were also reported [7].

The review identified several associations between Alarm Fatigue and safety-critical behaviors. These included mistrust of alarm systems and delayed responses [1, 7], as well as coping strategies such as reducing alarm volumes or disabling alarms altogether [40, 48].

From a patient perspective, our review suggests that Alarm Fatigue has significant repercussions. False alarms can delay HCPs’ response to actual emergencies, disrupting patient care and potentially leading to serious harm or even death [1, 40]. Patients are also affected in other ways, including disturbed communication with their families, impaired sleep [49], and overlooked signs of clinical deterioration [7]. Additionally, the constant noise from frequent alarms can negatively impact patients’ recovery, highlighting the need for more effective alarm management systems [49].

#### Strategies for reducing alarm fatigue

We categorized strategies to reduce Alarm Fatigue into seven areas: training programs [4, 7, 32, 39, 45], technological measures [1, 4, 7, 39, 41, 45, 50], clinical protocols [1, 4], device improvements [1, 4, 7, 39, 45], work environment enhancements [1, 4, 39, 45], preventive approaches [39], and additional strategies [1, 30, 39, 44]. These solutions are detailed in Supplementary Table 3 in the supplemental section.

##### Training programs:

Many solutions for reducing Alarm Fatigue have been proposed, with training programs playing a crucial role [7, 32, 39, 45]. Targeted, regular training (e.g. on alarm management) can help healthcare professionals better interpret and prioritize alarms, respond more effectively, and understand the operation of monitors, sensors, and alarms [7, 32].

##### Technological measures and device improvement:

Modern technologies can significantly reduce Alarm Fatigue [1, 7, 50]. AI-based systems can filter alarms and forward only relevant ones [1]. Smartphone-based systems could send alarms directly to the responsible staff, reducing unnecessary notifications [1, 50]. Existing equipment should also be optimized, such as improving sensor reliability and adjusting alarm thresholds to manageable levels [1, 7]. These technological solutions can lessen staff workload and improve response times to critical alarms [1, 4, 7, 39, 41, 45, 50].

##### Clinical protocols and assessment tools:

Implementing more effective clinical protocols or guidelines and using assessment tools can also help reduce Alarm Fatigue and support staff in responding more efficiently [1, 4]. Standardizing alarm limits and settings can decrease the number of false alarms, thereby reducing the burden on HCPs [1, 4].

##### Improving work environment and team coordination:

Enhancing the work environment and team coordination is another key aspect [4, 39, 45]. A well-organized and coordinated work setting can mitigate Alarm Fatigue by ensuring tasks are efficiently distributed and priorities are clearer [1, 4, 39, 45].

##### Preventive approaches:

Preventive measures, such as analyzing patient population risks, could also help reduce Alarm Fatigue [39]. Future strategies to reduce Alarm Fatigue could include the assessment and prediction of patient instability using machine learning approaches, as well as the planning and analysis of the alarm management process.

##### Additional measures:

Some comprehensive programs have been specifically designed to reduce Alarm Fatigue, these approached have been effective [30, 39]. Two such approaches identified in the literature include the “Smart Care” program, which incorporates both technological and non-technological measures [30, 39]. Technological aspects involve identifying causes and timely actions, personalized alarm settings, and reducing unnecessary alarms. Non-technological measures include effective teamwork, improving the physical environment, station arrangement, and self-soothing techniques [30]. Solet & Barach also developed an alarm management program offering numerous solutions for managing Alarm Fatigue [39].

### Additional key findings

The extracted additional key findings categorize the central results of alarm fatigue publications into six main areas: characteristics of alarm fatigue and alarms, response to alarms, impact on patient safety, effects on staff, differences between practice areas, and additional insights. Results are summarized in Table 3.

**Table 3:** Additional key findings

Main category	Key findings
1. Features of alarm fatigue and alarms	• Different levels of alarm fatigue • Frequent triggering of alarms • Many false and non-actionable alarms • Various main causes for alarm fatigue
2. Response to alarms	• More than half of the nurses disable or silence alarms • Delayed response • Underestimation of relevant alarms • Device alarms are less attended to during visiting hours
3. Effects of alarms on patient safety	• Missed signs of instability • Discrupted communication between patients and their families • Impaired sleep • Overlooked patient instability • Severe harm • Death
4. Effects of alarms on staff	• Overload • Decline in performance • Moderate to high levels of burnout • Impaired communication and concentration • Disorientation and distraction • Increased blood pressure and stress levels • Sleep disturbance and loss of sleep • Ear fatigue • Rule violations
5. Differences between practice areas	• Difference in alarm fatigue scores between practice areas • Different responses to alarms between practice areas
6. Additional findings	• Interruptions during high-priority task lead to increased alarm fatigue • Decreased perception of workload with an increased patient-to-nurse ratio • Difficulties using technological devices due to lack of training and inadequacies

## Discussion

**Fig. 3:**
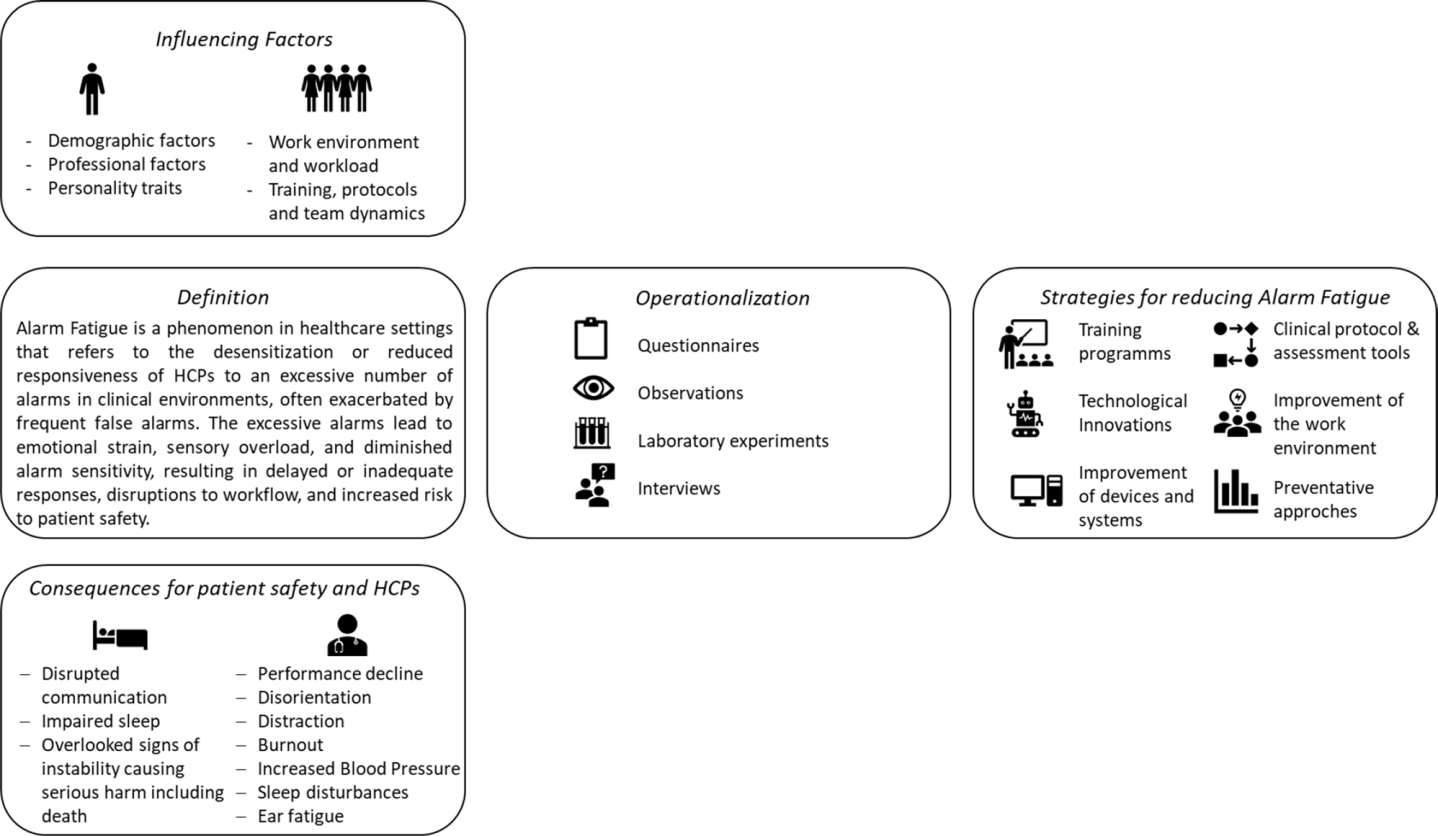
Graphical summary of the review’s objectives

The aim of this scoping review was to provide a comprehensive overview of Alarm Fatigue within the broader healthcare context based on existing literature. Specifically, we aimed to (1) propose a harmonized definition; (2) identify operationalization methods; (3) summarize influencing factors; (4) examine consequences; and (5) discuss potential strategies for reducing Alarm Fatigue (for a brief summary see Fig. 3).

### General insights

More than half of the publications (72%) included in this review were published in the last six years [4–6, 8, 9, 16–18, 22–36], indicating that Alarm Fatigue has gained prominence as a current issue. The global distribution of the publications suggests that Alarm Fatigue is a worldwide phenomenon. Most publications were primary research articles conducted in various healthcare settings [8, 16, 17, 22–28, 30–42], reflecting a strong interest in directly investigating Alarm Fatigue and its impacts in everyday clinical practice.

### Summary of evidence

#### Definition of alarm fatigue

The review aimed to summarize various definitions of Alarm Fatigue. All definitions recognize Alarm Fatigue’s serious consequences for patient safety and highlight its psychological impact on HCPs. Differences in definitions include varying causes of Alarm Fatigue, such as the high frequency of alarms [1, 23, 40] or the presence of false or non-actionable alarms [24, 37]. Based on the recurring themes identified across publications, we proposed a working definition of Alarm Fatigue (see Results section), which integrates key aspects such as desensitization, sensory overload, emotional strain, and impaired alarm responsiveness, ultimately increasing risk to patient safety. Given the lack of consensus in the literature, this definition should be regarded as a preliminary working proposal that synthesizes recurring patterns across existing studies. It offers a conceptual anchor to support the standardized operationalization of Alarm Fatigue, inform theoretical models, and guide the development of targeted interventions. Future research should critically examine, refine, and validate this definition to advance conceptual clarity and improve comparability across studies.

#### Operationalization of alarm fatigue

The review examined common methods for operationalizing Alarm Fatigue. Questionnaires, particularly “The Nurses’ Alarm Fatigue Questionnaire” by Torabizadeh et al., [46] are the most frequently used tool. This questionnaire is specifically designed for nurses, rather than all HCPs. Observational methods were employed in six studies to objectively capture HCPs behavior and reactions [15, 25, 35, 37, 38, 44]. Two studies used laboratory settings to simulate realistic scenarios and assess cognitive and physical load [31, 41], although such lab-based studies remain rare. There is a clear need for more laboratory-based studies to assess Alarm Fatigue in controlled environments, which could provide deeper insights into the underlying psychophysiological mechanisms. Furthermore, the absence of studies using physiological measurements to objectively assess Alarm Fatigue highlights a key research gap. Future studies employing such a multi-method approach would be critical for providing a more comprehensive understanding of Alarm Fatigue. Since the most effective measurement method has not yet been determined, combining different methods could provide a more comprehensive understanding. Laboratory studies on Alarm Fatigue are still rare. More lab studies are needed to understand the extent of Alarm Fatigue and its impact on HCPs, as well as to develop effective reduction strategies. Simulated hospital environments with training mannequins or virtual reality simulations could be used to create realistic conditions for such studies. Additionally, Ecological Momentary Assessments should be conducted before, during and after the introduction of new technologies aimed at reducing Alarm Fatigue. Biophysiological Measurements to objectively assess stress induced by Alarm Fatigue should be considered in future research. Furthermore, it is important to investigate differences between practice areas and professions to identify where HCPs may be more vulnerable to Alarm Fatigue. Additionally, more publications with homogeneous samples and balanced gender distribution are needed to explore age and gender differences in Alarm Fatigue.

#### Influencing factors

The review identified factors that may modify or moderate the relationship between alarm-related exposure (e.g., alarm frequency, false alarms) and the development of Alarm Fatigue. These influencing factors were categorized into two groups: individual characteristics and environmental conditions Although our review clearly highlights the need for more studies to better understand the exact influence of these factors on Alarm Fatigue in the context of a comprehensive etiological model, the existing studies provide strong evidence suggesting that behavioral and organizational prevention and intervention strategies, specifically addressing these factors, could serve as effective approaches to preventing Alarm Fatigue [1, 3, 8, 9, 23–26, 28, 35, 40].

In terms of individual factor, findings suggest potential moderating effects of demographic variables (i.e., age, gender) on Alarm Fatigue [9, 22–24, 28, 33, 35, 36, 40], but remain inconclusive especially with respect to gender [23, 28, 35]. These contradictions could particularly be due to varying gender distributions across samples. Regarding professional experience, the mixed findings suggest a dual role: while prolonged exposure may increase desensitization and risk of Alarm Fatigue, greater experience might also promote more effective alarm management and coping strategies. Higher education levels have been associated with lower Alarm Fatigue in some studies, possibly due to enhanced training and clinical knowledge, though these associations are not universally observed. Importantly, Alarm Fatigue has been reported across various clinical professions, including nurses and physicians, underscoring its relevance as a cross-professional issue. Psychological factors also appear relevant. Alarm Fatigue has been linked to elevated stress, emotional exhaustion, and depersonalization, suggesting a potentially bidirectional relationship that may create a reinforcing cycle of psychological strain and reduced responsiveness. Regarding personality traits, neuroticism has been associated with increased susceptibility to stress-related Alarm Fatigue, while conscientiousness and openness may offer protective effects through structured routines and adaptability. Extraversion and agreeableness, while linked to greater responsiveness to alarms, may also increase vulnerability to fatigue depending on contextual demands. These patterns point to the importance of individual dispositions in shaping alarm-related behavior and outcomes, an area that remains underexplored and could benefit from targeted psychometric approaches.

Environmental factors such as shift type, workload, and team dynamics yielded mixed results, reflecting the complexity of contextual influences on Alarm Fatigue [9, 23–26, 28] The impact of night shifts, for instance, may depend on institutional practices, alarm system design, and individual chronotypes. Inadequate training, unclear protocols, and poor communication were more consistently identified as contributing factors, highlighting modifiable targets for organizational interventions.

In sum, future research should use longitudinal and multi-level designs to clarify how influencing factors shape the relationship between alarm exposure and Alarm Fatigue. This is key to identifying underlying mechanisms and developing targeted interventions. Consequences for patient safety HCPs.

The results of our review indicate that Alarm Fatigue has far-reaching consequences. In relation to HCPs, chronic symptoms such as stress, exhaustion, and sleep disturbances not only impair individual well-being and performance but may also lead to increased sick leave. Given the essential role of HCPs in maintaining healthcare system functionality, such absences carry broader consequences for institutional resilience and societal healthcare delivery [51]. Moreover, the review highlights how Alarm Fatigue can undermine safety-critical behaviors including mistrust in alarm systems, delayed response times, and coping strategies such as lowering alarm volumes or disabling alarms entirely. While these behaviors may be intended to manage alarm overload, they can compromise alarm effectiveness and endanger patient safety. From the patient perspective, the consequences of Alarm Fatigue extend beyond these immediate risks. Disruptions in care continuity, impaired communication with family members, and disturbed rest due to frequent alarm noise can interfere with recovery [49].

Taken together, these consequences at multiple levels underline the urgency of addressing Alarm Fatigue through technical innovation, targeted training, organizational changes, and further research on alarm management and human factors in clinical settings.

#### Strategies for reducing alarm fatigue

Several strategies for reducing Alarm Fatigue were found and can be categorized into training programs, technological measures, clinical protocols, device improvements, work environment enhancements, preventative approaches and additional strategies. Technological interventions, particularly smart care solutions, appear promising, especially given that artificial intelligence is already being applied in clinical contexts for clinical decision support [46]. This is especially encouraging, as further advancements in this area are expected.

### Limitations

The aim of this scoping review was to capture the current knowledge on Alarm Fatigue through targeted literature research. The greatest strength of this review, in our opinion, lies in the fact that, unlike previous overviews on Alarm Fatigue, it does not focus on isolated aspects but, by addressing the definition, operationalization, influencing factors, impacts on patient safety and HCPs, and potential solution strategies in a single work, provides a comprehensive perspective on the topic. However, this review also has limitations, which will be outlined below.

First, in the absence of a universally accepted definition of Alarm Fatigue, the interpretation of our results should be approached with caution, as the publications varied significantly in the definitions they employed, or in some cases, did not specify how they defined Alarm Fatigue at all. We hope that by highlighting this gap and offering an initial proposal for a definition, we can contribute to the standardization of research in this field. Secondly, the review’s restriction to 32 publications limits the generalizability of the findings and as this is a scoping review there was no deeper evaluation of the quality or validity of individual studies. Furthermore, the literature search was confined to English-language publications, potentially overlooking relevant publications in other languages and countries. Most publications focused on nurses, so the impact of Alarm Fatigue on other professions like physicians or physiotherapists remains vague. Moreover, the results might be influenced by the publication bias, as only publicized studies were included in this review. This could result in a biased representation of the actual state of research.

## Conclusion

This scoping review provides a comprehensive and harmonized definition and conceptual framework of Alarm Fatigue, aiming to establish a shared understanding within the research and clinical community. It identifies methodologies to operationalize Alarm Fatigue and thoroughly examines the multifactorial influences contributing to its development. These include the interplay of psychosocial working conditions, such as workload, team dynamics, and organizational culture, alongside individual characteristics, such as gender, level of experience, personality traits, and pre-existing health conditions. Additionally, the review explores the multifaceted consequences of Alarm Fatigue, emphasizing its detrimental impact on patient safety and the physical and mental well-being of HCPs. Strategies to reduce Alarm Fatigue, as highlighted in the literature, are also discussed, underlining the need for targeted interventions at both the systemic and individual levels. The evidence suggests that while substantial progress has been made in understanding Alarm Fatigue, significant gaps remain, particularly concerning the conclusive identification of contributing factors and the effectiveness of proposed interventions. Addressing this critical issue requires a holistic, multifaceted approach that aligns with the harmonized definition and conceptualization proposed in this review, emphasizing its significance as a central area of ongoing research and practice improvement.

## Electronic supplementary material

Below is the link to the electronic supplementary material.

Supplementary Material 1

## Data Availability

No datasets were generated or analysed during the current study.

## References

[1] Cvach M. Monitor alarm fatigue: an integrative review. Biomed Instrum Technol. 2012;46(4):268–77. 22839984 10.2345/0899-8205-46.4.268

[2] Tanner T. The problem of alarm fatigue. Nurs Womens Health. 2013;17(2):153–7. 23594329 10.1111/1751-486X.12025

[3] Wilken M, Hüske-Kraus D, Klausen A, Koch C, Schlauch W, Hörig R. Alarm fatigue: Causes and effects. In: German medical data sciences: visions and bridges [Internet]. IOS Press; 2017 [cited 2024 Sep 26]. pp. 107–11. (Studies in health technology and informatics). Available from: https://ebooks.iospress.nl/doi/10.3233/978-1-61499-808-2-107 28883181

[4] Sowan A. Effective dealing with alarm fatigue in the intensive care unit. Intensive Crit Care Nurs. 2024;80:103559. 37801853 10.1016/j.iccn.2023.103559

[5] Chromik J, Klopfenstein SAI, Pfitzner B, Sinno ZC, Arnrich B, Balzer F et al. Computational approaches to alleviate alarm fatigue in intensive care medicine: A systematic literature review. Front Digit Health. 2022;4. 10.3389/fdgth.2022.843747 PMC9424650 36052315

[6] Li B, Yue L, Nie H, Cao Z, Chai X, Peng B, et al. The effect of intelligent management interventions in intensive care units to reduce false alarms: an integrative review. Int J Nurs Sci. 2024;11(1):133–42. 38352290 10.1016/j.ijnss.2023.12.008 PMC10859571

[7] Hravnak M, Pellathy T, Chen L, Dubrawski A, Wertz A, Clermont G, et al. A call to alarms: current state and future directions in the battle against alarm fatigue. J Electrocardiol. 2018;51(6 Suppl):S44–8. 30077422 10.1016/j.jelectrocard.2018.07.024 PMC6263784

[8] Lewandowska K, Mędrzycka-Dąbrowska W, Tomaszek L, Wujtewicz M. Determining factors of alarm fatigue among nurses in intensive care units—a Polish pilot study. J Clin Med. 2023;12(9):3120. 37176561 10.3390/jcm12093120 PMC10179395

[9] Shaoru C, Hui Z, Su W, Ruxin J, Huiyi Z, Hongmei Z, et al. Determinants of medical equipment alarm fatigue in practicing nurses: A systematic review. SAGE Open Nurs. 2023;9:23779608231207227. 37927965 10.1177/23779608231207227 PMC10621293

[10] Peters MDJ, Marnie C, Tricco AC, Pollock D, Munn Z, Alexander L, et al. Updated methodological guidance for the conduct of scoping reviews. JBI Evid Synth. 2020;18(10):2119–26. 33038124 10.11124/JBIES-20-00167

[11] Kordek M. Alert fatigue by other names: review of contributing fields regarding the ‘cry wolf’ effect. Oregon Health and Science University; 2013.

[12] Patel RS, Bachu R, Adikey A, Malik M, Shah M. Factors related to physician burnout and its consequences: A review. Behav Sci. 2018;8(11):98. 30366419 10.3390/bs8110098 PMC6262585

[13] Shanafelt TD, Balch CM, Bechamps G, Russell T, Dyrbye L, Satele D, et al. Burnout and medical errors among American surgeons. Ann Surg. 2010;251(6):995. 19934755 10.1097/SLA.0b013e3181bfdab3

[14] Tawfik DS, Phibbs CS, Sexton JB, Kan P, Sharek PJ, Nisbet CC, et al. Factors associated with provider burnout in the NICU. Pediatrics. 2017;139(5):e20164134. 28557756 10.1542/peds.2016-4134 PMC5404731

[15] Oliveira AECD, Machado AB, Santos EDD, Almeida ÉBD. Alarm fatigue and the implications for patient safety. Rev Bras Enferm. 2018;71(6):3035–40. 30517409 10.1590/0034-7167-2017-0481

[16] Dehghan M, Mokhtarabadi S, Rashidi E, Rahiminejad E, Asadi N. Correlation between professional quality of life and alarm fatigue symptoms among intensive care unit nurses. Health Sci Rep. 2023;6(10):e1583. 37822846 10.1002/hsr2.1583 PMC10563168

[17] Ding S, Huang X, Sun R, Yang L, Yang X, Li X, et al. The relationship between alarm fatigue and burnout among critical care nurses: A cross-sectional study. Nurs Crit Care. 2023;28(6):940–7. 37070292 10.1111/nicc.12899

[18] Nyarko BA, Yin Z, Chai X, Yue L. Nurses’ alarm fatigue, influencing factors, and its relationship with burnout in the critical care units: A cross-sectional study. Aust Crit Care. 2024;37(2):273–80. 37580238 10.1016/j.aucc.2023.06.010

[19] Tricco AC, Lillie E, Zarin W, O’Brien KK, Colquhoun H, Levac D, et al. PRISMA extension for scoping reviews (PRISMA-ScR): checklist and explanation. Ann Intern Med. 2018;169(7):467–73. 30178033 10.7326/M18-0850

[20] Peters MDJ, Godfrey CM, Khalil H, McInerney P, Parker D, Soares CB. Guidance for conducting systematic scoping reviews. JBI Evid Implement. 2015;13(3):141. 10.1097/XEB.0000000000000050 26134548

[21] Zotero [Internet]. Corprotation for digital scholarship. 2024. Available from: https://digitalscholar.org/.

[22] Ali Al-Quraan H, Eid A, Alloubani A. Assessment of alarm fatigue risk among oncology nurses in Jordan. SAGE Open Nurs. 2023;9:237796082311707. 10.1177/23779608231170730 PMC10134186 37124378

[23] Alkubati SA, Alsaqri SH, Alrubaiee GG, Almoliky MA, Alqalah TAH, Pasay-An E, et al. Levels and factors of nurses’ alarm fatigue in critical care settings in Saudi arabia: A multicenter cross-sectional study. J Multidiscip Healthc. 2024;17:793–803. 38410522 10.2147/JMDH.S452933 PMC10896094

[24] Bourji H, Sabbah H, Al’Jamil A, Khamis R, Sabbah S, Droubi N et al. Evaluating the alarm fatigue and its associated factors among clinicians in critical care units. Eur J Clin Med [Internet]. 2020 Dec 27 [cited 2024 May 7];1(1). Available from: https://www.ej-clinicmed.org/index.php/clinicmed/article/view/8

[25] Claudio D, Deb S, Diegel E. A framework to assess alarm fatigue indicators in critical care staff. Crit Care Explor. 2021;3(6):e0464. 34151285 10.1097/CCE.0000000000000464 PMC8205220

[26] Ergi̇n E, Bi̇Nay Yaz Ş, Atay A. Determining alarm fatigue among nurses in paediatric and adult intensive care units: A cross-sectional study. Turk Klin J Nurs Sci. 2023;15(2):341–8.

[27] Gündoğan G, Erdağı Oral S. The effects of alarm fatigue on the tendency to make medical errors in nurses working in intensive care units. Nurs Crit Care. 2023;28(6):996–1003. 37632222 10.1111/nicc.12969

[28] Ilter SM, Ovayolu O. Alarm fatigue and the factors that affect it in intensive care unit nurses: A cross-sectional study. Int J Caring Sci. 2023;16(3):1523–31.

[29] Lewandowska K, Weisbrot M, Cieloszyk A, Mędrzycka-Dąbrowska W, Krupa S, Ozga D. Impact of alarm fatigue on the work of nurses in an intensive care environment—a systematic review. Int J Environ Res Public Health. 2020;17(22):8409. 33202907 10.3390/ijerph17228409 PMC7697990

[30] Movahedi A, Sadooghiasl A, Ahmadi F, Vaismoradi M. Smart care for dealing with nurses’ alarm fatigue in the intensive care unit. J Nurs Scholarsh. 2023;55(4):825–33. 36631719 10.1111/jnu.12870

[31] Nagrecha S, Baldwin CL. The impact of false alarms on mental workload, stress and performance: implications for medical settings. Proc Hum Factors Ergon Soc Annu Meet. 2022;66(1):783–7.

[32] Nyarko BA, Nie H, Yin Z, Chai X, Yue L. The effect of educational interventions in managing nurses’ alarm fatigue: an integrative review. J Clin Nurs. 2023;32(13–14):2985–97. 35968774 10.1111/jocn.16479

[33] Regmi B, Shrestha B, Khanal S, Moktan S, Byanju R. Alarm fatigue among nurses working in critical care setting in a tertiary hospital, Nepal. Kathmandu Univ Med J KUMJ. 2023;21(81):28–32. 37800422

[34] Stiglich YF, Dik PHB, Segura MS, Mariani GL. The alarm fatigue challenge in the neonatal intensive care unit: A before and after study. Am J Perinatol. 2024;41:E2348–55. 37339673 10.1055/a-2113-8364

[35] Storm J, Chen H. The relationships among alarm fatigue, compassion fatigue, burnout and compassion satisfaction in critical care and step-down nurses. J Clin Nurs. 2021;30(3–4):443–53. 33174282 10.1111/jocn.15555

[36] Yahyaei S, Khoddam H, Alinaghimaddah S, Modanloo M. Prevalence of alarm fatigue and its relevant factors in critical care nurses: A cross-sectional study. J Res Dev Nurs Midw. 2023;20(1):20–3.

[37] Cho OM, Kim H, Lee YW, Cho I. Clinical alarms in intensive care units: perceived Obstacles of alarm management and alarm fatigue in nurses. Healthc Inf Res. 2016;22(1):46. 10.4258/hir.2016.22.1.46 PMC4756058 26893950

[38] Deb S, Claudio D. Alarm fatigue and its influence on staff performance. IIE Trans Healthc Syst Eng. 2015;5(3):183–96.

[39] Solet JM, Barach PR. Managing alarm fatigue in cardiac care. Prog Pediatr Cardiol. 2012;33(1):85–90.

[40] Casey S, Avalos G, Dowling M. Critical care nurses’ knowledge of alarm fatigue and practices towards alarms: A multicentre study. Intensive Crit Care Nurs. 2018;48:36–41. 29793861 10.1016/j.iccn.2018.05.004

[41] Cobus V, Meyer H, Ananthanarayan S, Boll S, Heuten W. Towards reducing alarm fatigue: Peripheral light pattern design for critical care alarms. In: Proceedings of the 10th Nordic Conference on Human-Computer Interaction [Internet]. Oslo Norway: ACM; 2018 [cited 2024 May 7]. pp. 654–63. Available from: 10.1145/3240167.3240218

[42] de Oliveira AEC, Machado AB, Santos EDD, de Almeida ÉB. Alarm fatigue and the implications for patient safety. Rev Bras Enferm. 2018;71(6):3035–40. 30517409 10.1590/0034-7167-2017-0481

[43] Lapeña JFF, Peh WCG. Various types of scientific articles. In: A guide to the scientific career [Internet]. John Wiley & Sons, Ltd; 2019 [cited 2024 Sep 26]. pp. 351–5. Available from: https://onlinelibrary.wiley.com/doi/abs/10.1002/9781118907283.ch37

[44] Stiglich YF, Dik PHB, Segura MS, Mariani GL. The alarm fatigue challenge in the neonatal intensive care unit: A before and after study. Am J Perinatol. 2023;a-2113-8364. 10.1055/a-2113-8364 37339673

[45] Li B, Yue L, Nie H, Cao Z, Chai X, Peng B, et al. The effect of intelligent management interventions in intensive care units to reduce false alarms: an integrative review. Int J Nurs Sci. 2023;11(1):133–42. 38352290 10.1016/j.ijnss.2023.12.008 PMC10859571

[46] Torabizadeh C, Yousefinya A, Zand F, Rakhshan M, Fararooei M. A nurses’ alarm fatigue questionnaire: development and psychometric properties. J Clin Monit Comput. 2017;31(6):1305–12. 27848141 10.1007/s10877-016-9958-x

[47] Satow L. Reliability and validity of the enhanced Big Five personality test (B5T) [Internet]. 2021 [cited 2024 Sep 26]. Available from: https://osf.io/wsugv

[48] ERCI Institute. Top 10 health technology hazards. 2012 p. 1–23. (Health devices). Report No.: 41(11). 23444722

[49] Hsu T, Ryherd E, Persson Waye K. Noise pollution in hospitals: impact on patients. J Clin Outcomes Manag JCOM. 2012;19:301–9.

[50] Chromik J, Klopfenstein SAI, Pfitzner B, Sinno ZC, Arnrich B, Balzer F et al. Computational approaches to alleviate alarm fatigue in intensive care medicine: A systematic literature review. Front Digit Health [Internet]. 2022 Aug 16 [cited 2024 May 6];4. Available from: https://www.frontiersin.org/articles/10.3389/fdgth.2022.843747/full 10.3389/fdgth.2022.843747 PMC9424650 36052315

[51] Dyrbye LN, Mayo Clinic, Sinsky CA, American Medical Association, Cipriano PF, American Nurses Association., Burnout among health care professionals: A call to explore and address this underrecognized threat to safe, high-quality care. NAM Perspect [Internet]. 2017 Jul 5 [cited 2024 Sep 25];7(7). Available from: https://nam.edu/burnout-among-health-care-professionals-a-call-to-explore-and-address-this-underrecognized-threat-to-safe-high-quality-care/

