# Supplementary material for: Alarm fatigue in healthcare: a scoping review of definitions, influencing factors, and mitigation strategies
Source: BMC Nurs. 2025 Jun 20;24:664. doi: 10.1186/s12912-025-03369-2 (PMC12181921; doi:10.1186/s12912-025-03369-2)
Supplement: Supplementary file 1 — Supplementary Material 1 [file 12912_2025_3369_MOESM1_ESM.docx]

Supplementary Tables (Tables longer than one A4 page)

**Supplementary Table 1**

*Search protocol with search strategy and number of results*

| No. | Database | Date | Search strategy | results |
| --- | --- | --- | --- | --- |
| 1 | PubMed | 19.04.2024 | "alarm fatigue" | 368 |
| 2 | CINAHL | 19.04.2024 | ("alarm fatigue") AND ("false alarm" OR "clinical alarms" OR "intensive care" OR "care" OR "hospital" OR "clinic" OR "nurses" OR "nursing" OR "staff" OR "measurement" OR "measuring" OR "impact" OR "performance" OR "staff performance" OR "psychological impact" OR "error" OR "influence" OR "influencing factors" OR "cause" OR "effect" OR "risk" OR "patient" OR "patient safety" OR "safety" OR "prevention" OR "implications" OR "reduction" OR "reduce" OR "decrease" OR "minimizing" OR "managing" OR "control" OR "solving" OR "intervention" OR "individual factors" OR "quality of professional life" OR "questionnaire" OR "review" OR "relevance" OR "monitor" OR "burnout" OR "response" OR "ward" OR "mental workload" OR "non-actionable") | 294 |
| 3 | MEDLINE | 19.04.2024 | ("alarm fatigue") AND ("false alarm" OR "clinical alarms" OR "intensive care" OR "care" OR "hospital" OR "clinic" OR "nurses" OR "nursing" OR "staff" OR "measurement" OR "measuring" OR "impact" OR "performance" OR "staff performance" OR "psychological impact" OR "error" OR "influence" OR "influencing factors" OR "cause" OR "effect" OR "risk" OR "patient" OR "patient safety" OR "safety" OR "prevention" OR "implications" OR "reduction" OR "reduce" OR "decrease" OR "minimizing" OR "managing" OR "control" OR "solving" OR "intervention" OR "individual factors" OR "quality of professional life" OR "questionnaire" OR "review" OR "relevance" OR "monitor" OR "burnout" OR "response" OR "ward" OR "mental workload" OR "non-actionable") | 123 |
| 4 | Google Scholar | 22.04.2024 | ("alarm fatigue") AND ("false alarm" OR "clinical alarms" OR "intensive care" OR "care" OR "hospital" OR "clinic" OR "nurses" OR "nursing" OR "staff" OR "measurement" OR "measuring" OR "impact" OR "performance" OR "staff performance" OR "psychological impact" OR "error" OR "influence" OR "influencing factors" OR "cause" OR "effect" OR "risk" OR "patient" OR "patient safety" OR "safety" OR "prevention" OR "implications" OR "reduction" OR "reduce" OR "decrease" OR "minimizing" OR "managing" OR "control" OR "solving" OR "intervention" OR "individual factors" OR "quality of professional life" OR "questionnaire" OR "review" OR "relevance" OR "monitor" OR "burnout" OR "response" OR "ward" OR "mental workload" OR "non-actionable") | The first 50 |
| 5 | Handsearch | 23.04.2024 | - | 11 |

**Supplementary Table 2**

*Overview of the 32 publications included*

| **Citation** | **Definition of Alarm Fatigue** | **Sample** | **Operationalization of AF** | **Considered additional factors** | **Study Design** | **Key findings** |
| --- | --- | --- | --- | --- | --- | --- |
| Ali Al-Quraan H, et al.(2024) | No direct definition  “The definition of fatigue is the inability to act. There are two types of fatigue: acute, which occurs after a rest period, and chronic, which occurs after a long period of rest (Winwood et al., 2005). A monitoring device produces excessive stimulation, leading to alarm fatigue (Sendelbach, 2012).” | 222 nurses, 60.4% female, 25.18±3.33 years old | the nurses’ alarm fatigue questionnaire (Torabizadeh et al., 2017) | age, gender, educational level, working area*, and years of experience in nursing | Cross-sectional design | Total mean alarm fatigue score: 31.62 (7.15) (scale ranging from 0 to 52) |
| Alkubati et al. (2024) | “Alarm Fatigue occurs when medical professionals are overexposed to alerts, it can cause sensory overload and lead to missed and desensitized alarms.6 It may negatively affect the nurses’ quality of care as well as the patient safety.” | 298 nurses, 57% males, mean age of 31.7±5.7, | Nurses’ Alarm Fatigue Questionnaire (Torabizadeh et al., 2017) | Age*, sex*, marital status, nationality*, years of experience*, level of training, department, job position, patient-to-nurse ratio, shift length, number of working hours per shift*, and additional outside employment | Cross-sectional design | total mean score of alarm fatigue was 26.38±8.30 out of 44 |
| Bourji et al. (2020) | “The Alarm Fatigue (AF) occurs when clinicians are exposed to a large number of false alarms which can cause alarm desensitization.” | 337 participants, 77.2% of participants were nursing staff and 22.8% were physicians, mean age was 30.79 (SD=7.26; median=29) years, 61.4% were women | Alarm fatigue questionnaire (Torabizadeh et al., 2017) | age, gender*, marital status, educational level*, job position*, type of hospital*, work unit*, years of overall experience in work, years of overall ICU experience*, nurses-to-patient-ratio, monitoring equipment and alarm management*, rate of non-actionable alarms*, types of medical devices and alarms* | cross-sectional design | Total mean alarm fatigue score: 30.57 (7.89) (scale ranging from 0 to 44)  identified explanatory factors |
| Carcereri de Oliveira et al. (2018) | “Alarm fatigue is the implication of a resistance/desensitization in the perception of alarm signaling. […] When a large number of alarms are activated at the same time, suppressing other clinically significant alarms and allowing some relevant ones to be silenced, disabled or ignored by health professionals, the occurrence of alarm fatigue is verified. Absence of responses to relevant signals suggests impairment on safety and worsens the clinical conditions of patients in intensive therapy” | Adult ICU healthcare team: Two nurses, ten technicians, two doctors, and two physiotherapists. | Response time after alarm noise (and general observation of health professionals’ conduct) | Professional category, number of alarms attended in percent, Mean response time, physiological variables on part of the patients which produced the alarms (heart rate; arterial pressure; peripheral oxygen saturation; end of continuous pump infusion; peak pressure; positive airway pressure; expiatory flow; loose Electrode), electromedical device which triggered the alarm, conduct of health care professionals right before the alarm | observational and descriptive research with quantitative approach | 66.03% of the alarm activation equipment were never attended by any professional for more than 10 minutes  Nursing was the professional category that reacted the most (31.06%) while other professionals within the multidisciplinary team only responded to 2.91% of the alarms. |
| Casey et al. (2018) | No direct definition  “Nurses have become overwhelmed by the sheer volume of alarms leading to alarm apathy” | 250 registered nurses from six hospital sites, 166 of those responded, 89% were women. 93% staff nurses, 7% clinical nurse managers, 62% had 11 years or more critical care experience | The Health Technology Foundation (HTF) 2011 Clinical Alarms Survey (CAS) (Funk et al., 2014) (adapted for the Irish population) | Education level, professional experience, knowledge of alarm fatigue, knowledge of preventing alarm fatigue, perception of alarms, alarm related patient adverse events, trust in alarms, non-actionable, staffing, hospital model, critical care beds, capacity, training/improvement initiatives | descriptive cross-sectional design | All hospitals reported patient adverse events related to clinical alarms. A significant 90% of nurses experienced frequent non-actionable alarms, which disrupted patient care for 91% and diminished trust in alarm systems for 81%. While 52% of nurses were unsure how to prevent alarm fatigue, those who felt knowledgeable tended to customize alarm parameters more often. Only 31% believed that alarm management policies were effectively utilized. Additionally, nurses identified staffing shortages and frequent alarms as major obstacles to improving alarm management. |
| Cho et al. (2016) | “Alarm fatigue occurs when medical staff are overwhelmed by excessive clinical alarms; in particular, false (positive) alarms, inappropriate alarms-setting ranges, and the overuse of patient monitors act as principal factors that cause alarm fatigue. Of these, the most problematic factor is false alarms: frequent false alarms may produce the 'cry wolf' effect and may cause nurses to regard significant alarms as false and thus fail to respond properly.” | 77 ICU nurses, mean age: 29.4 (±5.8) years; mean career in ICUs: 6.1 (±4.8) years; 97,4% were female | Eight relevant items from the table of subjective symptoms of fatigue revised by the Japanese Occupational Hygiene Association in 2002 and from the instrument applied by Kim and Sung | Age, gender, education level, work area, current position, working experience at ICUs, experience of hazards in patient safety related to clinical alarms, number of alarms, cause of alarm, alarm-generating device, alarm-setting status of patient monitors and mechanical ventilators, nurses' alarm recognition (14 items), obstacles to effective alarm management (9 items), | Descriptive design | During 48 hours of observation, 63.8% of alarms were categorized as false alarms. 72.2% of the false alarms from patient monitors were due to technical issues.  The alarm fatigue score was 24.3 ± 4.0 (out of 35)  In only 9 out of 48 cases (18.8%) nurses set an alarm range that reflected the patient’s conditions |
| Chromik et al. (2022) | “Alarm fatigue occurs as a result of an overwhelming amount of alarms. This sensory overload can lead to alarm desensitisation and a loss of competence of the ICU staff (physicians and nurses) in dealing with alarms, ultimately resulting in patient harm or even death” | - | -  (To the best of the authors’ knowledge, there is no standard measure of alarm fatigue available.) | Included publications per year, alarm types (Heart rate related, Arterial blood pressure alarms, Oxygen saturation alarms, Respiration-related, Other), type-specific and alarm-agnostic solutions, types of computational approaches used (software and hardware), use of signal quality indicators (SQIs), prediction of patient deterioration, use of “super alarm pattern”, novel methods of alarm presentation | systematic literature review | Alarm fatigue can be reduced through IT-based solutions. Most solution stragies focus on: HR-related alarms, arrhythmia-related alarms  There is little work on preventing non-actionable alarms, likely due to a lack of validated datasets on this topic. |
| Claudio et al. (2021) | “The frequency of nuisance or “nonactionable” alarms can lead to “sensory overload” and “desensitization” in caregivers, potentially making them unwilling to respond to real threats. Experts have defined this sensory overload and desensitization as alarm fatigue.” | 24 ICU professionals (18 nurses, 6 unit clerks) | subjective workload assessment technique (SWAT) and subjective measures of affect (desensitization): boredom, apathy, distrust | job title, years of experience, working unit, working facility, alarm-related information (how often does nuisance alarms occur? how noisy is the environment? Is it possible to differentiate the alarm sounds and identify the sources from among others? perception of alarm utility, emotional response), age, education, experience working in the ICU, previous experience participating in an alarm management study, alarm criticality, task priority, patient-to-staff ratio, time elapsed since the start of the shift, sound level, extroversion, conscientiousness, neuroticism, agreeableness, openness, | standardized clinical alarm survey and an observational study | Statistically significant factors influencing SWAT as well as boredom, apathy and distrust include: work-related factors (patient-to-staff ratio, time elapsed since the beginning of the shift, and task priority) and personality factors (neuroticism, agreeableness, and extroversion).  Nurses were more likely to exhibit indicators of alarm fatigue when: alarms sound during high priority tasks, high patient-to-staff ratio and if nurses had been on their shift for a longer time. Neuroticism correlates positively with alarm fatigue indicators, openness, agreeableness and extraversion correlate negatively. |
| Cobus et al. (2018) | “Alarm fatigue is a known effect in this demanding working environment which means a desensitization as well as a delayed response time for alarms.” | A feasibility study: 11 participants including 6 women, normal or corrected vision, aged between 18-41 years  A participatory design study: 10 participants including 6 women, normal or corrected vision, aged between 18-33 years | -  (alarm fatigue was not directly measured) | error rate in perceiving/identifying light patterns, subjective ratings of: pattern perceptibility, pattern distraction, pattern urgency, pattern comfort/pleasantness, informativeness of patterns, intuitiveness of patterns, associations/interpretations of light patterns, task performance during pattern presentation (error rate, response time, low vs. high priority alarm vs. technical alarm, both vs. one eye, physical and cognitive load, color | A feasibility study and a participatory design study | A head-mounted display effectively presents information within the user’s peripheral vision.  Specific light patterns are optimal for indicating different alarm priorities: short blinking represents high-priority red alarms, blue light patterns are perceived as less urgent compared to red or yellow patterns. |
| Cvach (2012) | “Alarm fatigue occurs when clinicians are barraged by so many false or nonactionable alarm signals that they become desensitized. False alarms occur when there is no valid triggering event, whereas nonactionable alarms correctly sound but for an event that has no clinical relevance.” | - | - | five main themes of studies were found: excessive alarms and effects on staff; nurse's response to alarms; alarm sounds and audibility; technology to reduce false alarms; and alarm notification systems  further factors in the areas: alarm management, education of staff, and documentation | Integrative Review | Most nurses report alarms are too frequent, disruptive to care, and reduce confidence, leading to alarm disabling.  Excessive alarm noise can be an occupational hazard and impede patient recovery.  Hospital noise levels consistently exceed WHO recommendations, with a trend of increasing noise since 1960. |
| Deb & Claudio (2015) | “According to most of the qualitative definitions of alarm fatigue, it has been found that staff get overwhelmed and desensitized due to a high number of false alarms and continuous beeping monitors, resulting in alarm fatigue.” | Six employees and 18 registered nurses: | Subjective Workload Assessment Technique (SWAT)  National Aeronautics and Space Administration-Task Load Index (NASA-TLX).  Affect: (boredom, apathy, and distrust) | Gender, age, Education, Experience (years), Experience at ICU (years), Specialized training on alarm monitoring, experience of participating in alarm related research, total number of alarms, staff to patient ratio (workload), time elapsed since start of the shift, alarm type, alarm criticality, noise level, task priority, staff personality, shift (day/night), staff ID, patient deaths, damage to patient conditions or extended stays among other accidental events, response to alarms (yes/no), response time, number of ignored alarms | Observational study | 84.6% of participants reported frequent nuisance alarms that disrupt patient care.  These nuisance alarms contribute to reduced trust in alarm systems, leading 53.8% of caregivers to disable alarms (with 46.2% remaining neutral) |
| Dehghan et al. (2023) | No direct definition  “Nurses may not appropriately respond to alarms because of the increasing frequency of these alarms in various medical devices. Complications such as headache, fatigue, and stress following repeated alarms cause nurses to ignore alarms, set alarms in a distant place regardless of the physiological needs of patients or even interrupt these alarms.” | 196 nurses with a bachelor’s or higher degree in nursing, at least six months of work experience in an intensive care unit, no self-reported hearing impairments, and no psychological disorders. | alarm fatigue symptom (AFS) scale (Dehghan et al.) | gender, age, marital status, education level, type of employment, work experience in ICU, secondary employment, compassion satisfaction, burnout, secondary trauma stress, psychosomatic symptoms, care‐related symptoms | cross‐sectional design | majority of ICU nurses reported: Moderate levels of compassion satisfaction, burnout, secondary traumatic stress, mild symptoms of alarm fatigue  Alarm fatigue symptoms were significantly correlated with: negatively correlation with compassion satisfaction, positively correlations with burnout and secondary traumatic stress  Alarm fatigue emerged as a significant predictor for all three professional quality of life subscales.  Additional predictors of burnout included: work experience in the ICU, having a second job |
| Ding et al. (2023) | “Alarm fatigue refers to a phenomenon whereby health care providers are desensitized to alarms and alerts when working in a clinical environment.” | 236 nurses from five hospitals, working as registered nurses in an intensive care unit (82.6% were women, 63.6% were married and all were aged between 21-30 years. | Nurses' Alarm Fatigue Questionnaire (Torabizadeh et al., 2017) | Age (Years), Gender, Marital status, Education*, Types of ICU*, Professional title, Employment types*, Working years, Specialist nurse, Night shift, Clinical teacher, Shift types, Experience of PSIs, Burnout (MBI)* | descriptive-analytical cross-sectional study design | The findings indicated that critical care nurses reported moderate levels of alarm fatigue, with an average score of 21.11 ± 6.83 (out of 44)  Most nurses exhibited moderate to high levels of emotional exhaustion, depersonalization, and reduced personal accomplishment. |
| Ergİn et al. (2023). | “The Emergency Care Research Institute (ECRI) defines alarm fatigue as the emotional pressure medical staff face when they are exposed to too many alarm sounds. Alarm fatigue is a phenomenon that affects nurses when they work in a clinical environment where alarm sounds are frequently heard.” | 166 nurses working at universities and public hospitals (aged 18 or older; working in either a pediatric or adult intensive care unit). | Nurses' Alarm Fatigue Questionnaire (Torabizadeh et al., 2017) | sex, age, marital status, educational status, length of service in nursing, the department worked in, type of work schedule, length of service in critical care, and average weekly working hours*, features of the noise meters used in the units* | descriptive and cross-sectional study | Nurse’s sociodemographic characteristics did not influence the level of alarm fatigue  Increased weekly working hours and the absence of noise measurement devices lead to more alarm fatigue among ICU nurses  Nurses felt overwhelmed by an excessive number of tasks and a continuous stream of clinical alerts |
| Gündoğan & Erdağı Oral (2023) | “Alarm fatigue is defined as a per-son's unresponsiveness to alarms with excessive sensory overload asa result of exposure to a large number of alarm sounds.” | 382 nurses working in intensive care units. | Alarm Fatigue Scale (Kahraman et al., 2020) | age, gender, marital status, educational background, work experience, length of service in the unit, working clinic, and weekly working hours, Tendency to make medical mistakes (measured with the Medical Error Tendency Scale in Nursing)* | cross-sectional and correlational study | Alarm signals are suspected to contribute to fatigue and insensitivity to alarms among nurses, this could increase their tendency to make medical errors by leading to increased difficulties in concentrating and inattentiveness among nurses |
| Hravnak et al. (2018) | “Alarm fatigue occurs when non-actionable alarms are in the majority, and clinicians develop decreased reactivity, causing them to “tune out” or ignore the alarms.” | - | - | Frequency of alarms, alarm accuracy and reliability, alarm desensitization, cognitive overload in healthcare providers, customization and personalization of alarm systems, technological innovations and alarm integration, organizational and policy factors, patient safety and outcomes | review | Excessive clinically irrelevant alarms strain clinicians and the care system, risking patient safety by leading to overlooked signs of instability  Non-actionable alarms stem from various technical, patient-related, clinical, and organizational factors, contributing to alarm fatigue along the monitoring care continuum  This fatigue can disrupt communication between patients and families, impair sleep, and result in missed signs of patient instability  As the number of non-actionable alarms increases, clinician response delays also rise |
| Ilter & Ovayolu (2023) | “Alarm fatigue is defined as “the fatigue, sensory overload, boredom, reluctance to hear, and depersonalization in parallel with exhaustion after some time” occurring when the nurse is exposed to loud and different alarms for longer times” | 121 ICU nurses | Structured questionnaire, Nurses’ Alarm Fatigue Questionnaire (Torabizadeh et al., 2017) | Age*, gender, marital status*, educational level, length of work in the profession*, way of working*, working time in ICUs*, ICU worked in, type of technological device*, suitability of devices, effectiveness of devices, difficulty using devices*, reason for difficulty | cross-sectional study | alarm fatigue among ICU nurses was moderate and linked to age, marital status, profession, and time spent in ICU  the technological devices used by nurses in their workday and the duration of their use impacts alarm fatigue  the majority of intensive care nurses believed that the use of technology-based devices affects care, but they had difficulties using these devices due to insufficient training and inadequacies of the device |
| Lewandowska et al. (2023) | “The absence of a filter to identify repeating, insignificant and/or false alarms before healthcare professionals are alerted can contribute to the sensory overburdening of medical staff, which is known as alarm fatigue.” | 400 Intensive Care Unit nurses. Mean age 35.7 ± 10.0 years. The majority of participants were women (88.0%), had a Master of Science in Nursing (60.0%), worked 12 h shifts (76.7%). | Nurses’ Alarm Fatigue Questionnaire (Torabizadeh et al., 2017) | Gender, Education, Specialization in “Anesthesiology nursing and intensive care”, Type of ward, the structure of ward, Shift length, the ward has alarm management systems for monitoring devices, Participation in training programs related to use of monitoring devices available in the ward* | cross-sectional design | total mean score of alarm fatigue was 25.8±5.8 (out of 44) |
| Lewandowska et al. (2020) | “In the case of alarm fatigue, it is defined as an excessive exposure to the stimulus generated by the monitoring unit (Sendelbach, 2012).” | - | - | Frequent false alarms, which lead to reduced attention or response to alarms when they occur, difficulty in understanding the priority of an alarm, Inadequate staff to respond to alarms as they occur, difficulty in hearing alarms when they occur, difficulty in identifying the source of an alarm, overreliance on alarms to call attention to patient problems, noise competition from non-clinical alarms and pages, lack of training on alarm systems, difficulty in setting alarms properly. | Systematic review | 90-98% of nurses report that nuisance alarms are frequent and disrupt patient care.  Up to 85-99% of alarms are false or clinically insignificant.  69.4% of nurses in one study reported experiencing moderate to high levels of alarm fatigue, while only 31-58% knew about proper alarm management procedures |
| Li et al. (2024) | “Alarm fatigue is a common phenomenon in hospitals, which causes nurses to feel nervous or stressed and distrustful of the alarm system, thus delaying a response, ignoring, muting, or turning off alarms.” | - | - | Sensitivity to alarms, Changes in patient conditions, the usefulness of alarm notification system, Alarm load disturbs workflow, Ignore alarms in an urgent situation, Nuisance alarms disrupt patient care, Workload | Integrative review | Most smart alarm management systems had positive effects: reductions in the total number of alarms, the duration of alarms, and the response time of nurses to alarms, as well as a reduction in alarm fatigue among nurses. |
| Movahedi et al. (2023) | “The frequency of exposure to alarms especially non-actionable ones desensitizes clinical staff to alarms and leads to inadequate responses to vital alarms during the provision of care. This critical problem is known as alarm fatigue and has negative impacts on the quality and safety of care (Cvach, 2012; Horkan, 2014; Sendelbach & Funk, 2013)” | 18 nurses consisting of 6 men and 12 womenworking in 12 ICUs. Mean age of 33 years and an average work ex-perience of 8 years in the ICU. | in- depth, semi-structured, individual interviews  (no concrete measure for alarm fatigue) | Age, Work experience in the intensive care unit (ICU), Gender, Education level, Type of ICU | Qualitative design | Nurses' smart care involved various proactive interventions, focusing on six subcategories: identifying causes and taking timely actions, personalizing alarm settings, reducing unnecessary alarms, fostering effective teamwork, enhancing the physical environment, and promoting self-calmness. These strategies aim to mitigate alarm fatigue and decrease physical and mental strain on nurses |
| Nagrecha & Baldwin (2022) | no direct definition  “False alarms (FAs) refer to alarms which do not require a response, such as those caused by movement artifacts. An overabundance of FAs can lead to desensitization and alarm fatigue (Kristensen, Edworthy, & Ozcan,2016).” | Thirty-six university students (76.3% women, Mean age = 22.18 years, SD age = 5.034) | - NASA TLX questionnaire  - A dual-task method (to mirror demands placed on nurses) > error rate and response time  - N-back task (working memory) > error rate and response time | The number of interruptions, whether the task being performed has a series of steps with a clear order of progression (structured) or not (unstructured), high and low false alarm rate during n-back task | within-subject design | Participants made significantly more false responses in the high false alarm (FA) condition (average 3.80) compared to the low FA condition (average 1.72).  Starting with a low-false alarm condition initially improved both speed and accuracy in the subsequent high-false alarm condition across tasks. |
| Nyarko et al. (2023) | “Alarm fatigue develops when nurses are overburdened by the sheer volume of alarm sounds which could lead to alarm desensitization and, consequently, results in missed alarms or a delayed response to alarms (Schmid et al., 2011).” | - | - | - | Integrative review (9 pretest– post-test designs, 1 RCT, 2 quasi- experimental and 1 descriptive study design) | Most studies found that educational interventions effectively reduced the total number of alarms and false alarms |
| Nyarko et al. (2023) | “Alarm fatigue as defined by the American Association of Critical Care Nurses is a sensory overload that happens when clinicians are faced with or exposed to an excessive number of alarm alerts, which can bring about desensitization to alarm sounds and a high rate of missed alarms.” | 364 nurses (60% female, 40% male, mean age: 28.73 ± 3.73 years) | Alarm Fatigue Questionnaire (Yin, 2021)  Maslach Burnout Inventory (Maslach & Jackson, 1981) | Age*, gender, educational level*, years working in critical care units*, working unit, years working as a nurse*, professional title, alarm training, policies on alarm management*, headaches due to alarms, anxiety* and stress* | Cross-sectional design | Alarm fatigue scores comparable to those in China, Korea or Lebanon, indicating that alarm fatigue is a global health concern that persists all over the world  Multiple regression analysis revealed that years of experience in the critical care unit, the specific unit of work, alarm management policies, and levels of anxiety and stress accounted for 12% of the overall variance in nurses' alarm fatigue  Most critical care nurses reported high burnout in the Emotional Exhaustion (EE) component |
| Regmi et al. (2023) | “[Alarm fatigue] occurs when nurses become overwhelmed by the total number of alarm signals which can result in alarm desensitization and eventually contributes to missing of serious and important changes in a patient’s condition, thus failing to respond properly.” | 56 Nurses. More than half of respondents were younger than 25 years, about three-fourth were working in an adult intensive care unit | a single center, cross-sectional survey, Nurses’ Alarm Fatigue Questionnaire (Torabizadeh et al., 2017) | Age, marital status, educational level, current working area, working experiences in nursing profession, working experience in critical care area, knowledge about alarm fatigue, training/inservice education on alarm management, responsible for alarm limit setting in their unit, experience of adverse patient events related to the clinical alarm problems | cross-sectional design | overall mean of alarm fatigue among nurses: 28.03 ± 12.813 (out of 44)  66.1% of nurses were familiar with the concept of alarm fatigue, but only 7.1% had training on alarm management.  There was no significant correlation between alarm fatigue and the selected socio-demographic and work-related factors |
| Shaoru et al. (2023) | “Medical equipment alarm fatigue refers to a phenomenon where medical personnel who are exposed to alarms from multiple medical equipment for a long time become less sensitive to these alarms, resulting in the potential for them to ignore critical equipment alarms. […] (Wilken et al., 2017).” | a total sample size of 2,848 nurses. | The clinical alarm fatigue scale (one study used the Japanese version of said scale) | Educational level, Time of shift*, working years, professional title*, gender, amount of training, knowledge about alarm fatigue, alarm parameter setting, false alarm rate | Systematic review (14 cross-sectional studies) | Alarm fatigue among clinical nursing staff was reported at a moderate level (21.76 (95% CI [20.27, 23.25]) out of a possible 44)  Risk factors for alarm fatigue in clinical nurses: night shifts, different job titles, a high false alarm rate and excessive monitoring alarms |
| Solet & Barach (2012) | “Alarm fatigue occurs when the sheer number and frequency of monitor alarms becomes overwhelming, leading to delayed or failed responses, and even the disabling and silencing of alarms.” | - | - | Clinician knowledge, skills, and abilities, Cognitive biases, Risk preference, State of health, Fatigue (including sleep deprivation, circadian effects, Breaks and boredom, Substance use/abuse (e.g., alcohol hangover effects), Stress, Personality factors, Task distribution, Task demands, Workload, Job burnout, Shift-work, Teamwork/team dynamics, Interpersonal communication (clinician–clinician, clinician–patient), Interpersonal influence, Groupthink, Noise, Lighting, Temperature and humidity, Motion and vibration, Physical constraints (e.g., crowding), Distractions, Device usability, Alarms and warnings, Automation, Maintenance and obsolescence, Protective gear), Production pressure, Culture of safety (vs. efficiency), Policies, Procedures, Documentation requirements, Cross coverage, Hierarchical structure, Reimbursement policies, Training programs | Review article | Noisy healthcare facilities lead to impaired communication and concentration, disorientation, distraction, increased blood pressure and stress levels, getting used to noises, rule violations (such as switching off alarms), sleep disturbances, loss of sleep  - False alarm rates are reported to be as high as 83-85%.  - Background noise levels in hospitals typically range from 45-68 dB(A), with peaks exceeding 85-94 dB(A).  - In one study, 77% of nurses agreed that "nuisance alarms" disrupt patient care.  - In another study, 78% of nurses said nuisance alarms reduce trust in alarms and cause caregivers to disable them. |
| Sowan (2024) | No direct definition  “According to nurses, alarm fatigue is complex, triggered by excessive false alarms, reduces trust in alarm systems, and results in adverse events.” | - | Alarm number was used as a proxy for alarm fatigue | The eight dimensions of the sociotechnical model:  - Hardware and software  - Clinical content  - Human-computer interface  - People  - Workflow and communication  - Internal organizational features  - Measurements and monitoring | Integrative review | Many individual and bundled interventions effectively reduced the number of alarms with varying degrees, most common interventions focused on the clinical content aspects of the sociotechnical model, given the complexity of alarm system safety demands, a systematic approach to assessment, management, and evaluation is essential |
| Stiglich et al. (2023) | “The term alarm fatigue (AF) among caregivers refers to the desensitization that occurs when exposed to a large number of sounds while working.” | 200 patient observations in the Neonatal Intensive Care Unit (NICU) of the Hospital Italiano de Buenos Aires | Observation of three factors: Proportion of nonactionable alarms, response time to alarms and proportion of true alarms | alarm type*, response time*, shift, characteristics of the patient when the alarm activation event took place, gestational age (GA), birthweight (BW), need of respiratory support, vasoactive drugs, and/or total parenteral nutrition (TPN), and the health caregiver that responded to the alarm (physician, nurse, or respiratory therapist). | cross-sectional design | the implementation of an Alarm Management Program (AMP) effectively reduced the number of non-actionable alarms and decreased the response time to alarms. |
| Storm & Chen (2021) | “Desensitisation to monitor alarms is known as alarm fatigue (Cvach, 2012). Nurses who care for critically ill patients may endurea greater number of alarms and may experience alarm fatigue alongwith compassion fatigue or burnout (Petersen & Costanzo, 2017).” | 52 registered nurses, 82% were women (n=43), 40,4% were 21–30 years old, 25% were 31–40 years, 7,7% were 41–50 years, 25% were 51–60 years | direct observation by designated observers: response time  Alarm fatigue was coded as 0 for no alarm fatigue (response time ≤ 10 minutes) and 1 for alarm fatigue (response time > 10 minutes). | Gender*, Age*, Race, Nursing Unit*, Nurse-to-Patient Ratio*, Shift worked, Average number of hours worked per week, years of experience, compassion fatigue, burnout, compassion satisfaction | A correlational and predictive quantitative study | 40% of the participants exhibited signs of alarm fatigue (n = 21) whereas 59.6% (n = 31) participants did not show alarm fatigue |
| Wilken et al. (2017) | “The term “Alarm fatigue” is commonly used to describe the effect which a high number of alarms can have on caregivers: Frequent alarms, many of which are avoidable, can lead to inadequate responses, severely impacting patient safety.” | - | - | Consumables (materials), Architecture (Milleu), Infrastructure (Machine), Configuration (Management), Workflow (Method), Team (Man) | Scoping review | Alarm fatigue has causes in diverse areas: technology and technical infrastructure, workflow/process, ICUs as a socio-technical system and the people who work in the team; Potential effects of unnecessary alarms: continuous stress, unnecessary work, frequent disruption of care processes, acute cognitive overload, desensitization of staff, "noise pollution" and patient disruption |
| Yahyaei et al. (2023) | “Alarm fatigue occurs when healthcare providers, especially nurses, are exposed to numerous alarms of varying importance, leading to gradual desensitization and actions such as turning off the alarm system, delaying a response, or not responding.” | 308 eligible nurses. The mean age of the nurses was 30.7±6.54 years, 79.2% of the nurses were female | Nurses’ Alarm Fatigue Questionnaire (Torabizadeh et al., 2017) | age (year), gender*, marital status, educational level*, habitat, type of units, Working experiences in clinical setting (year), working experiences in critical care units (year), work shifts, overtimes type*, overtime per month (hours), level of alarm fatigue n (%), income, working place (hospital), employment status | descriptive-analytical design | the mean score for nurses’ alarm fatigue was 24.1 (SD = 6.52). Only a small percentage (1.9%, n=6) did not report any alarm fatigue, 26% experienced mild fatigue (n=132), 63.3% had moderate fatigue (n=161), and 8.8% exhibited severe fatigue (n=9). |

* indicates significant associations with Alarm Fatigue

**Supplementary Table 3**

*Recommendations for Reducing Alarm Fatigue*

| Main categories | Examples | Sources |
| --- | --- | --- |
| 1.Training programms | - Training to understand the functionality of monitors, sensors, and alarms - Trainings - Educational interventions - Training in configuration and threshold setting - Alarm training and education | Hravnak et al., 2018; Li et al., 2024; Nyarko et al., 2023; Solet & Barach, 2012; Sowan, 2024 |
| 2.Technological innovations | - Devices that counteract acoustic strain, high cognitive load, and the risk of Alarm Fatigue in ICUs - Use of SuperAlarm patterns - „D.A.S.H." system that adjusts alarm volume to background noise to avoid unnecessary noise pollution - phone-based systems - Self-developed apps or software - Placement of central monitors to improve sightlines and minimize unnecessary response distances - Smartphone notification to a specific staff member instead of all nearby personnel - Alarm system that sends alarms to different people and different devices at different times - Alarm system that sends alarms to different people and different devices at different times - Implementation of a secondary device notification system - Sending cardiac monitoring alarms to a separately confirmed pager - Alarm delay - Notification delay - Development of more advanced device algorithms - Smartphone-based system for cardiac monitoring using PPG (Photoplethysmogram) - Peripheral light alarm signals - Use of artificial intelligence - Implementation of signal filtering - Wireless technologies - Animated steps on monitoring devices | Chromik et al., 2022; Cobus et al., 2018; Cvach, 2012; Hravnak et al., 2018; Li et al., 2024; Solet & Barach, 2012; Sowan, 2024 |
| 3.Improvement of devices and systems | - Improvement of sensor reliability - Threshold changes - Adjustment and expansion of alarm parameters - Changing the alarm tone to vibration mode - Improving alarm design for the end user - Adapting alarms to the needs of the patients - Setting activated alarms to manageable limits and levels | Cvach, 2012; Hravnak et al., 2018; Li et al., 2024; Solet & Barach, 2012; Sowan, 2024 |
| 4. Clinical protocols and assessment tools | - Implementation of more effective clinical protocols or guidelines - Documenting alarm parameters in the patient’s record | Cvach, 2012; Sowan, 2024 |
| 5. Improvement of the work environment and team coordination | - Interdisciplinary collaboration - Improved acoustic environments - Adequate patient-to-nurse ratios - Improved team coordination - Multidisciplinary teamwork - Use of noise reduction strategies | Cvach, 2012; Li et al., 2024; Solet & Barach, 2012; Sowan, 2024 |
| 6. Preventive approaches | - Risk analysis of patient populations | Solet & Barach, 2012 |
| 7. Additional measures | - "Smart Care" (Identifying the cause and acting in a timely manner, personalized alarm settings, reducing the number of unnecessary alarms, effective teamwork, improving the physical environment, ward layout, self-soothing) - Investigation of indicators for patient condition deterioration - Development of scenarios to assess the effectiveness of training specific skills - "Alarm Management Program" (Improvement of the acoustic environment, surveys among healthcare providers, alarm configuration and threshold setting, improvement of alarm algorithms, optimization of medical devices, improvement of the "Source-Path-Receiver" model, alarm standards and regulations, design and manufacturing of medical devices, improvement of alarm management, training recommendations) - Skin preparation and regular replacement of ECG leads and electrodes - Simulation with medical training mannequins such as SimMan 2G or ECG-enabled Resusci-Anne - Design of simulation scenarios based on reported damages or near misses with triggers | Cvach, 2012; Movahedi et al., 2023; Solet & Barach, 2012; Stiglich et al., 2023 |
